# Maternal seafood consumption and fetal growth: a birth cohort study in urban China

**DOI:** 10.1186/s12884-023-05431-w

**Published:** 2023-04-13

**Authors:** Zhang Wei, Wang Li, Cao Lei, An Caixia, Zhang Chuan, Wang Jianqin

**Affiliations:** 1 Lanzhou University Second Hospital, No. 82, Cui Yingmen, Linxia Road, Chengguan District, Lanzhou City, 730050 Gansu Province China; 2 Gansu Provincial Maternity and Child Care Hospital, 143 North Road Qilihe District, Lanzhou, 730050 Gansu Province China

**Keywords:** Seafood consumption, Fetal growth, Low birth weight, Cohort study, China

## Abstract

**Background:**

Seafood is a good source of essential fatty acids which has a presumably beneficial effect on developing embryos and fetuses, although it is also a source of contaminants. In this context, pregnant women are faced with conflicting reports on the risk and benefits of seafood consumption. This study aims to assess whether the consumption of seafood during pregnancy was associated with fetal growth in an inland city in China.

**Methods:**

This study included 10,179 women who delivered a singleton live birth in Lanzhou, China. Seafood consumption was assessed using a Food Frequency Questionnaire. Maternal data including birth outcomes and maternal complications information is extracted from the medical records. Associations between seafood consumption and fetal growth indicators were analyzed using multiple linear regression and multiple logistic regression.

**Results:**

There was a positive association between total seafood consumption and birth weight (*β* = 0.027, 95%*CI*:0.030–0.111) but no association concerning birth length or head circumference. Seafood consumption was associated with decreased risk of low birth weight (*OR* = 0.575, 95% *CI*: 0.480, 0.689). The frequency of seafood consumption during pregnancy showed a trend toward a positive association with low birth weight. Significantly reduced rates of low birth weight were found in women who consumed more than 75 g of seafood/week during pregnancy as compared to women with no or very low intakes (*P* for trend 0.021). A significant interaction was observed between pre-pregnancy BMI and seafood consumption on birth weight among underweight women, but not among overweight women. Gestational weight gain partially mediated the association between seafood consumption and birth weight.

**Conclusions:**

Maternal seafood consumption was associated with decreased risk of low birth weight and increased birth weight. This association was mainly driven by freshwater fish and shellfish. These results further corroborate the present dietary recommendation to the Chinese Nutrition Society for pregnant women, especially those with underweight pre-pregnancy BMI and inadequate GWG. In addition, our findings provide implications for future interventions to improve seafood consumption among pregnant women to prevent low birth weight babies in the inland city in China.

## Introduction

Seafood, defined as marine and freshwater fish and shellfish, has been well-demonstrated to contain nutrients that maintain and promote health [1]. The past few decades have seen a gradually increasing trend of seafood consumption all over the world due to technological advancement in processing, distribution, transportation, and storage [2, 3]. Globally, the annual seafood consumption per capita has increased from almost 10 kg in 1960 to over 20 kg in 2014, and seafood contributed to over 20% of animal protein for more than 3.1 billion people [4]. Significant differences exist in the seafood consumption patterns in different countries and between the inland and coastal regions [2]. For instance, Asia consumed two-thirds of the world’s seafood, with 33.6 million tons in China, and 36.9 million tons outside China [5].

Abundant evidence has consistently shown that seafood consumption during pregnancy is associated with favorable fetal growth and birth outcomes. A recent literature review and meta-analysis concluded that increased seafood consumption was associated with a reduced risk of low birth weight (LBW), small for gestational age (SGA), and preterm birth (PTB) [6]. Seafood contains a diversity of nutrients that are beneficial for fetal growth and development, including n-3 long-chain polyunsaturated fatty acids (n-3 LCPUFAs), iodine, protein, selenium, and vitamins A, D, and E [7]. In particular, n-3 LCPUFAs, such as docosahexaenoic acid (DHA) and eicosapentaenoic acid (EPA), play an essential role in promoting fetal growth through a shift of the prostacyclin/thromboxane A balance, reduced blood viscosity, and increased placental blood flow [8].

However, substantial studies have also indicated that seafood may be a potential source of chemical pollutants such as polychlorinated biphenyls (PCB), dioxins, and methyl mercury (Hg), which may lead to adverse birth outcomes [8, 9]. In particular, Hg is among the top ten chemicals of concern worldwide and its major source is seafood [9]. Hg exposure in pregnancy can cause a series of pregnancy complications including spontaneous miscarriage, premature birth, congenital disability, and fetus developmental problems [10, 11]. Additionally, maternal Hg exposure may also cause neurobehavioral dysfunctions in children leading to poor academic performance in listening, reading, writing, etc. [10, 11].

The inconsistent findings on the association between maternal seafood consumption and fetal growth may arise from the differences in the types of seafood assessed, which may contain different types and levels of nutrients and pollutants [12]. It is thus suggested that an evidence-based risk-benefit assessment of seafood is necessary to guide the recommendation of maternal seafood consumption [1]. However, most of the previous studies were conducted in coastal cities of developed countries with high seafood consumption, and the question remained as to whether such effects could be observed in inland cities of developing countries with low seafood consumption. Results from European and US studies may not generalize to the Chinese inland cities such as Lanzhou city where seafood consumption is less common. The dietary guidelines for pregnant women of the Chinese Society of Nutrition recommend that seafood should be consumed 350–525 g per week during the second and third trimesters of pregnancy [13]. Yet few pregnant women in Lanzhou City achieved this standard for seafood consumption. It is thus important to investigate the effect of seafood consumption on fetal growth in Lanzhou City to guide future dietary interventions and recommendations.

The objective of the present study was to examine the association between seafood consumption and fetal growth (birthweight, birth length, and head circumference) and to determine the importance of the type of fish in this association. In addition, we also examined how seafood consumption during pregnancy affects the risk of low birth weight. In a secondary analysis, we examined whether the association between maternal seafood consumption and fetal growth varied by pre-pregnancy BMI, and whether gestational weight gain (GWG) mediated the association between seafood consumption and birth weight.

## Methods

### Population and study design

**Fig. 1:**
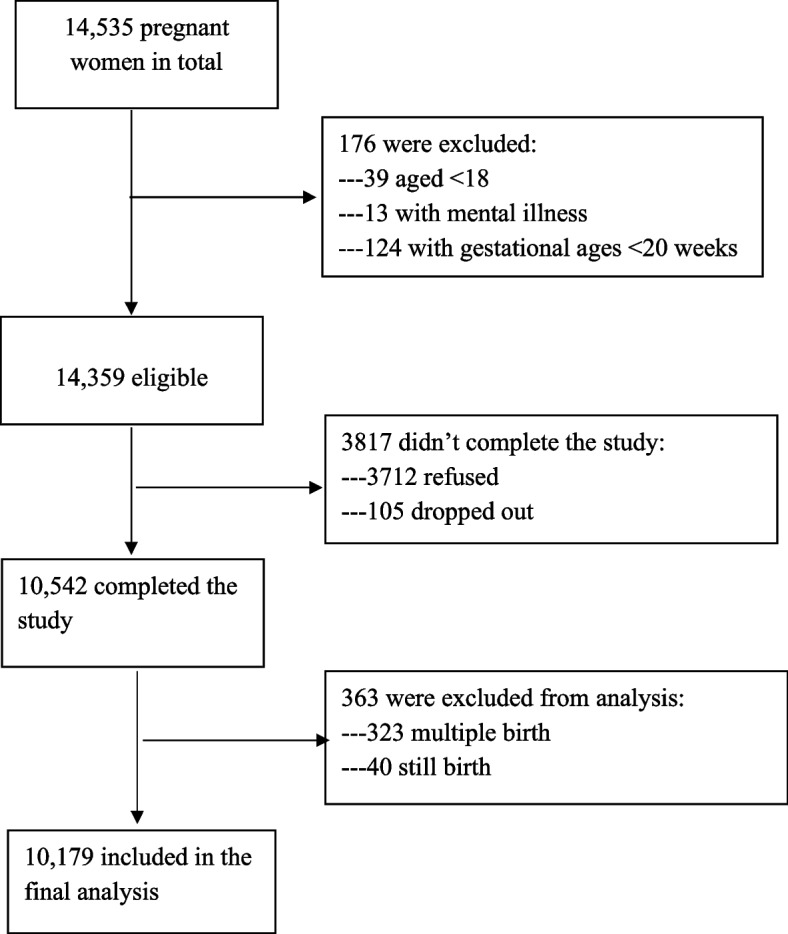
Flow diagram of the participants recruitment

The birth cohort study was conducted from 2010 to 2012 in the Gansu Provincial Maternity and Child Care Hospital (GSMCH), the largest maternity and childcare hospital in Lanzhou, China [14]. Inclusion criteria included: (1) aged 18 years or older, (2) with no history of mental illness, and (3) with gestational ages ≥20 weeks. A total of 14,535 pregnant women came to the hospital for delivery during the study period, among who 14,359 women were eligible after excluding 39 women aged under 18 years old, 13 women with mental illness, and 124 women with gestational ages of under 20 weeks. All eligible women were invited to participate in the study to complete in-person interviews and structured questionnaires. Among the 14,359 pregnant women approached for study participation, 3712 refused and 105 dropped out, leading to a total of 10,542 pregnant women completing the study with a response rate of 73.4%. After the exclusion of multiple births and stillbirths, 10,179 women having singleton live birth were included in the final analysis (Fig. 1).

All study procedures were approved by the human investigation committees at the Gansu Provincial Maternity and Child Care Hospital (No. 2010GSFYIRB09). Each eligible woman who came to the hospital for delivery was explained the study’s purpose and procedure in detail. Those who agreed to participate in the study were invited to participate in face-to-face interviews within 1–3 days after delivery after providing written informed consent. A standardized and structured questionnaire was used to collect information on demographic, reproductive and medical history, smoking behavior, alcohol, occupational and residential history, physical activity level, and seafood consumption during pregnancy. On the other hand, information on birth outcomes and maternal complications was extracted from medical records.

### Seafood consumption

Seafood consumption was assessed using a Food Frequency Questionnaire (FFQ) that inquired about the dietary habits of a wide range of various food during pregnancy. The FFQ contains 33 items assessing 33 types of various food, among which 3 items were about seafood, including freshwater fish (carp, etc.), saltwater fish (hairtail, etc.), and shellfish (shrimp, crabs, mussels, etc.). For each seafood item, participants were asked about both the frequency and the amount (g) of consumption. For each item, participants need to circle whether they eat on a daily, weekly, or monthly basis, followed by how many times (direct estimated numbers) and how much quantity (assessed by portion size images) they eat under each circled category based on their choice. Seafood consumption frequency was further classified into four categories: “never “, “≤1 time a week “, “2 -3 times a week”, and “≥4 times a week”. Seafood consumption quantity was further classified into five categories by quartiles: “never”, “< 14 g/week”, “14–75 g/week”, “76–176 g/week” and “> 176 g/week”.

### Outcome variables

Infant birth weight (g), birth length(cm), and head circumference(cm) measures were extracted from medical records. Gestational age was established based on the date of the last menstrual (LMP) self-reported by the mothers. However, in the case of a discrepancy between LMP and an early ultrasound measurement of ≥7d, the latter was used to determine gestational age. Low birth weight was defined as any newborn with a birth weight < 2500 g [15]. Macrosomia was defined as an estimated fetal weight of more than 4000 g [16]. Small for gestational age (SGA) was defined as a weight below the 10th percentile for gestational age, while large for gestational age (LGA) was defined as a birth weight (BW) greater than the 90th percentile for age [17].

### Other variables

Maternal height and pre-pregnancy weight were used to compute maternal body mass index (BMI, measurement, kg/m^2^) before pregnancy, which was categorized as underweight (BMI < 18.5 kg/m^2^), normal weight (18.5 kg/m^2^ ≤ BMI < 24 kg/ m^2^), and overweight and obese (BMI ≥ 24 kg/m^2^) using the standard of Working Group on Obesity in China [18]. The list of covariates for the multivariate models included maternal age (< 25, 25–29, 30–34, ≥ 35 years), years of education (< 9, 10–15, ≥16), monthly income(RMB < 2000, 2000–4000, > 4000), parity (nulliparous or parous), gestational diabetes mellitus (yes or no), gestational hypertension(yes or no), physical activities during pregnancy (physical activities no < 30 min at ≥3 times/week), passive smoke during pregnancy, multi-vitamin and protein supplement intake during pregnancy (yes or no, collected from the FFQ) and total energy intake (continuous variable).

### Statistical analysis

Statistical significance was assessed with the chi-square statistic for all categorical variables. Associations between seafood consumption and continuous variables such as birth weight, birth length, and head circumference were analyzed using multiple linear regression. Associations between seafood consumption and categorical variables such as low birth weight, macrosomia, SGA, and LGA were analyzed using multiple logistic regression. We used linear regression to calculate P for trend when examining seafood intake across the ordered categories and Student’s t-test for dichotomous variables. In addition, all regression models were adjusted for potential confounders of maternal characteristics. The variance inflation factor test for multicollinearity was well within limits, indicating that all confounders in the model could reliably assess their independent contribution. All *P* values were two-sided and defined to be significant at *P* < 0.05. All statistical analyses were performed with IBM SPSS Statistics for Macintosh (Version 25.0, Armonk, NY: IBM Corp).

## Results

### Descriptive statistics

Consumption of any seafood was reported by 82.9%(*n* = 8441) of women, while 17.1%(*n* = 1738) of women reported no seafood consumption in our study. In the total sample, the median energy intake calculated by the FFQ was 1613·94 kcal, with the 25th and 75th percentile being 1347 kcal and 1906 kcal, respectively. Table 1 shows a comparison of maternal characteristics by seafood consumption status. Compared to those who never consumed seafood, those who ever consumed seafood were generally older, with longer gestational age, with higher education, with higher income, and less likely to be parous. Women consuming seafood were more likely to have physical activities, take multivitamins and protein supplements during pregnancy, and less likely to have pregnancy hypertension and preterm birth.

**Table 1:** Comparison of maternal characteristics by seafood consumption status

Maternal characteristics	Seafood consumption		
	Ever (***n*** = 8441)	Never (***n*** = 1738)	
	N (%)	N (%)	*P*
**Age, years**			
**< 25**	**1215 (14.4)**	**419 (24.1)**	**< 0.001**
**25–29**	**4159 (49.3)**	**696 (40.0)**	
**30–34**	**2302 (27.3)**	**416 (23.9)**	
**≥ 35**	**765 (9.1)**	**207 (11.9)**	
**Gestational age, weeks**			
**< 37**	**733 (8.7)**	**286 (16.5)**	**< 0.001**
**37–42**	**7663 (90.7)**	**1438 (82.7)**	
**≥ 42**	**45 (0.6)**	**14 (0.8)**	
**Education, years**			
**≤ 9**	**1536 (18.2)**	**702 (40.4)**	**< 0.001**
**10–15**	**3400 (40.3)**	**577 (33.2)**	
**≥ 16**	**3399 (40.3)**	**380 (21.9)**	
**Missing**	**106 (1.3)**	**79 (4.5)**	
**Monthly income (CNY)**			
**< 2000**	**1770 (21.0)**	**651 (37.5)**	**< 0.001**
**2000–4000**	**4159 (49.3)**	**624 (35.9)**	
**> 4000**	**2302 (27.3)**	**227 (13.1)**	
**Missing**	**737 (8.7)**	**236 (13.6)**	
**Parity**			
**Nulliparous**	**6281 (74.4)**	**1068 (61.4)**	**< 0.001**
**Parous**	**2160 (25.6)**	**670 (38.6)**	
**Pre-pregnancy BMI**			
**< 18.5**	**1741 (20.6)**	**285 (16.4)**	**0.059**
**18.5–23.9**	**5640 (66.8)**	**1079 (62.1)**	
**≥ 24**	**903 (10.7)**	**182 (10.5)**	
**Missing**	**157 (1.9)**	**192 (11.0)**	
**Active smoking during pregnancy**			
**No**	**8372 (99.2)**	**1722 (99.1)**	**0.667**
**Yes**	**69 (0.8)**	**16 (0.9)**	
**Passive smoking during pregnancy**			
**No**	**6847 (81.1)**	**1390 (80.0)**	**0.271**
**Yes**	**1594 (18.9)**	**348 (20.0)**	
**Alcohol drinking during pregnancy**			
**No**	**8424 (99.8)**	**1734 (99.8)**	**0.810**
**Yes**	**17 (0.2)**	**4 (0.2)**	
**Physical activities**			
**No**	**1217 (14.4)**	**407 (23.4)**	**< 0.001**
**Yes**	**7224 (85.6)**	**1331 (76.6)**	
**Multi-vitamin intake during pregnancy**			
**No**	**6570 (77.8)**	**1509 (86.8)**	**< 0.001**
**Yes**	**1871 (22.2)**	**229 (13.2)**	
**Protein supplement intake during pregnancy**			
**No**	**8325 (98.6)**	**1725 (99.3)**	**0.034**
**Yes**	**116 (1.4)**	**13 (0.7)**	
**Gestational diabetes**			
**No**	**8354 (99.0)**	**1722 (99.1)**	**0.676**
**Yes**	**87 (1.0)**	**16 (0.9)**	
**Pregnancy hypertension**			
**No**	**8068 (95.6)**	**1587 (91.3)**	**< 0.001**
**Yes**	**373 (4.4)**	**151 (8.7)**	
**Preterm birth**			
**No**	**7708 (91.3)**	**1452 (83.5)**	**< 0.001**
**Yes**	**733 (8.7)**	**286 (16.5)**	
**Baby sex**			
**Male**	**4465 (52.9)**	**909 (52.3)**	**0.651**
**Female**	**3976 (47.1)**	**829 (47.7)**	

*Abbreviation*: *BMI* Body mass index

### Associations between seafood consumption and fetal growth

Table 2 shows the associations between seafood consumption (total and by type) with the following continuous variables of fetal growth indicators: birth weight, birth length, and head circumference while controlling for all potential confounders of maternal characteristics. Total seafood consumption was significantly positively associated with birth weight (β =0.027, 95%CI: 0.030–0.111, *P* < 0.001) but not with birth length or head circumference. When examining such associations by seafood type, both freshwater fish (β =0.061, 95%CI: 0.014–0.107, *P* < 0.001) and shellfish (β =0.019, 95%CI: 0.009–0.069, *P* < 0.001) consumption were significantly positively associated with birth weight, while saltwater fish consumption was not associated with any fetal growth indicators.

**Table 2:** Associations between seafood consumption with birth weight, birth length, and head circumference of infant born

	Birth weight z-value		Birth length		Head circumference	
	β	95%CI	β	95%CI	β	95%CI
**Total of seafood**	**0.027**	**0.030–0.111**	**0.012**	**−0.007-0.031**	**−0.007**	**−0.230-0.100**
**Types of seafood**						
**Freshwater fish**	**0.061**	**0.014–0.107**	**0.007**	**−0.006-0.021**	**−0.002**	**−0.014-0.009**
**Saltwater fish**	**0.012**	**−0.007-0.056**	**− 0.013**	**−0.030-0.004**	**− 0.009**	**−0.023-0.004**
**Shellfish**	**0.019**	**0.009–0.069**	**0.009**	**−0.005-0.023**	**0.001**	**−0.011-0.013**

Adjusted for maternal age, educational level, monthly family income, parity, gestational age, baby sex, hypertensive disorder during pregnancy, gestational diabetes, pre-pregnancy BMI, smoking (active and passive smoking) during pregnancy, physical activities during pregnancy, multi-vitamin and protein supplement intake during pregnancy and total energy intake

Table 3 shows the associations between seafood consumption with the following categorical variables of fetal growth indicators: low birth weight, macrosomia, SGA, and LGA while controlling for all potential confounders of maternal characteristics. Significant associations were observed between seafood consumption with low birth weight (β = 0.575, 95%CI:0.480–0.689, *P* < 0.001) and SGA (β = 0.590, 95%CI:0.501–0.696), but not with macrosomia or LGA. Compared to those who never consumed seafood, those who consumed seafood had decreased risk of low birth weight by 42.5% and SGA by 41%.

**Table 3:** Associations between seafood consumption with low birth weight, macrosomia, SGA and LGA

	Total seafood consumption			
	Never	Ever		
	N	N	OR (95%CI)	*P*
**Low birthweight**	**227**	**494**	**0.575 (0.480–0.689)**	**< 0.001**
**Macrosomia**	**75**	**423**	**1.024 (0.971–1.327)**	**0.855**
**SGA**	**89**	**176**	**0.590 (0.501–0.696)**	**< 0.001**
**LGA**	**15**	**92**	**1.065 (0.892–1.273)**	**0.484**

Adjusted for maternal age, educational level, monthly family income, parity, gestational age, baby sex, hypertensive disorder during pregnancy, gestational diabetes, pre-pregnancy BMI, smoking (active and passive smoking) during pregnancy, physical activities during pregnancy, multi-vitamin and protein supplement intake during pregnancy and total energy intake

*Abbreviation*: *SGA* Small for gestational age, and *LGA* Large for gestational age

**Fig. 2:**
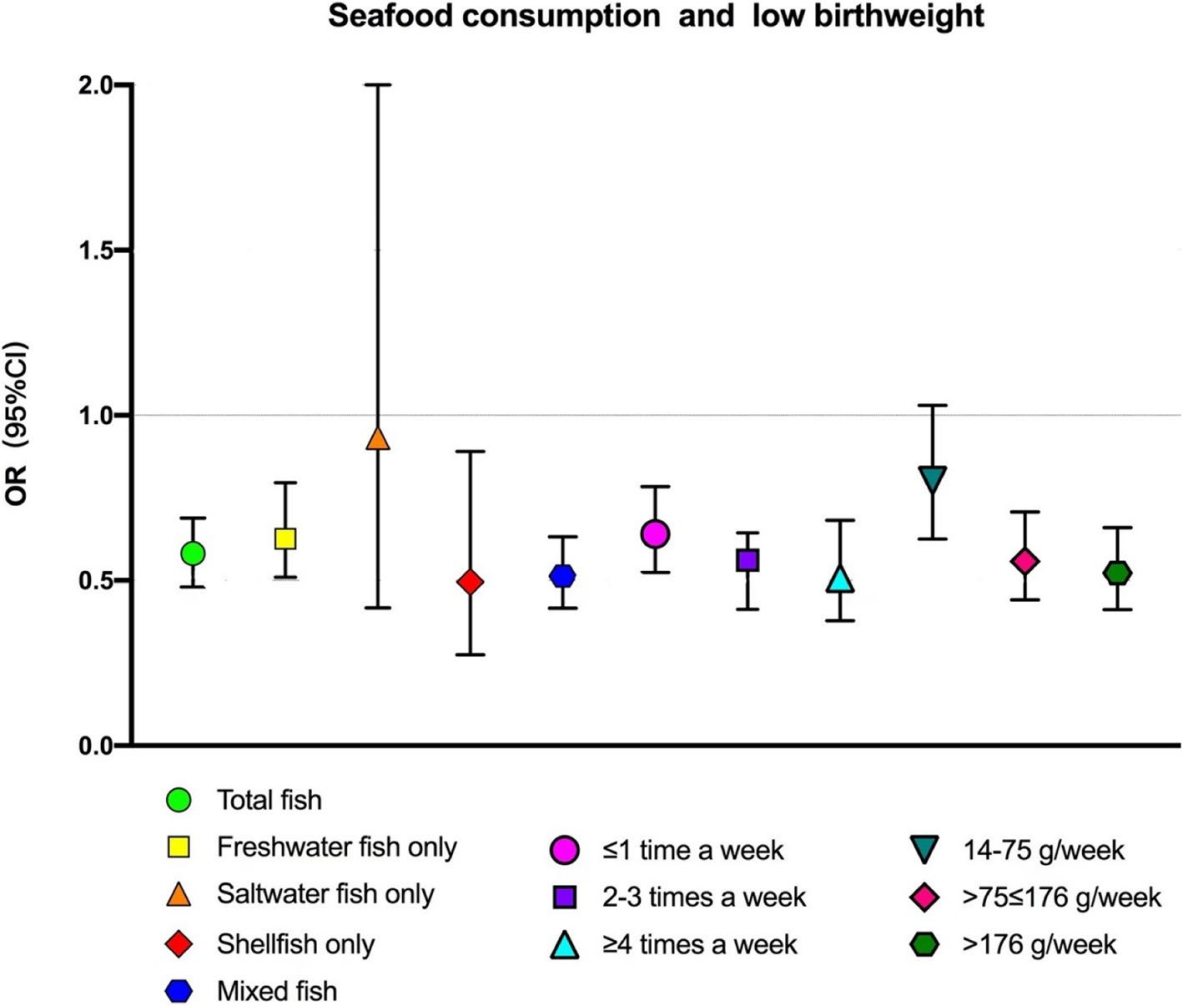
Associations between seafood consumption and low birthweight by seafood consumption type, frequency, and quantity. Adjusted for maternal age, educational level, monthly family income, parity, gestational age, baby sex, hypertensive disorder during pregnancy, Gestational diabetes, pre-pregnancy BMI, smoking (active and passive smoking) during pregnancy, physical activities during pregnancy, multi-vitamin and protein intake during pregnancy and total energy intake

Figure 2 shows the associations between seafood consumption and birthweight by seafood consumption type, frequency, and quantity while controlling for all potential confounders of maternal characteristics. Seafood consumption was associated with decreased risk of low birth weight, reflected in freshwater fish, shellfish intake, and mixed fish (any two or three kinds of fish) intake, but not saltwater fish. Increased frequency of seafood consumption was also associated with decreased risk of low birth weight, with a significant trending effect (*P* < 0.001). Compared to women consuming no seafood, the risk of low birth weight decreased by 35.9% among women consuming ≤1 time a week (OR = 0.641, 95% CI:0.524–0.784), by 48.4% among women consuming 2–3 times a week (OR = 0.516,95%CI:0.413–0.644), and by 49.3% among women consuming ≥4 times a week (OR = 0.507, 95%CI:0.378–0.682). In addition, an increased quantity of weekly seafood consumption was associated with a decreased risk of low birth weight, with a significant trending effect (*P* < 0.001). Compared to women consuming < 14 g/week, the risk of low birth weight decreased by 44.2% among women consuming 76–176 g/week (OR = 0.558,95%CI:0.441,0.707) by 47.8% among women consuming > 176 g/week (OR = 0.522,95%CI:0.412,0.660). No significant association was observed between the 14–75 g/week consumption group with the risk of low birth weight.

### Seafood consumption and low birthweight by pre-pregnancy BMI and GWG

Considering the important effect of pre-pregnancy BMI and GWG on birth weight, we further tested the interaction between pre-pregnancy BMI and seafood consumption on birth weight, as well as the mediation effect of GWG on the association between seafood consumption on birth weight in our secondary analysis. The results of the interaction analysis were shown in Table 4. Seafood consumption was associated with decreased risk of low birth weight in both women with underweight pre-pregnancy BMI (OR = 0.656, 95%CI: 0.434–0.993) and women with overweight pre-pregnancy BMI (OR = 0.445, 95%CI: 0.224–0.886), but not in women with normal pre-pregnancy BMI. We found a significant interaction effect of pre-pregnancy BMI in the association between seafood consumption and fetal growth in the pre-pregnancy BMI underweight group (*P* = 0.008), but not in the overweight group (*P* = 0.802). Additionally, further mediation analysis showed a partial mediation effect of GWG on the association between seafood consumption and infant birth weight. Increased seafood consumption was associated with increased GWG, which in turn, was associated with increased birth weight. The indirect effect of GWG on the association between seafood consumption and infant birth weight accounted for 11.8% of the total effect (*p* < 0.05).

**Table 4:** Associations between total seafood consumption and low birth weight by pre-pregnancy BMI (*n* = 9830)

Fish consumption		Low birthweight	
	N	N	OR (95%CI)
**Pre-pregnancy BMI underweight**			
**Never consumed fish**	**294**	**36**	**1**
**Ever consumed fish**	**1780**	**114**	**0.656 (0.434–0.993)**
**Pre-pregnancy BMI normal**			
**Never consumed fish**	**1070**	**134**	**0.894 (0.592–1.349)**
**Ever consumed fish**	**5606**	**312**	**0.522 (0.355–0.767)**
**Interaction** ***p*** **value**			**0.008**
**Pre-pregnancy BMI overweight**			
**Never consumed fish**	**182**	**30**	**1**
**Ever consumed fish**	**898**	**55**	**0.445 (0.224–0.886)**
**Pre-pregnancy BMI normal**			
**Never consumed fish**	**1070**	**134**	**1.016 (0.543–1.900)**
**Ever consumed fish**	**5606**	**312**	**0.655 (0.360–1.194)**
**Interaction** ***p*** **value**			**0.802**

Adjusted for maternal age, educational level, monthly family income, parity, gestational age, baby sex, hypertensive disorder during pregnancy, gestational diabetes, pre-pregnancy BMI, smoking (active and passive smoking) during pregnancy, physical activities during pregnancy, multi-vitamin and protein supplement intake during pregnancy and total energy intake

## Discussion

In this cohort study, we found higher consumption of seafood had a beneficial effect on low birth weight, which was mainly driven by freshwater fish and shellfish consumption. No association between seafood consumption and birth length or head circumference was observed in our population. Significantly reduced rates of low birthweight were found in women who consumed more than 75 g of seafood/week during pregnancy as compared to women with no or very low intakes. Also, significant interactions between pre-pregnancy BMI and seafood consumption on birth weight were found among the underweight group, but not in the overweight group. GWG partially mediated the association between seafood consumption and birth weight.

Our study demonstrated a positive association between maternal seafood consumption and increased birth weight, which is consistent with previous epidemiological studies showing that increased seafood intake was associated with increased fetal growth measures [19–25]. These findings have reasonable biological mechanisms. The n-3 LCPUFAs widely existent in seafood promote the shift of the prostacyclin/thromboxane A balance to a more antiaggregatory and vasodilator state, which may increase the placental flow and, as a consequence, fetal growth [26]. When we examined seafood consumption by type, significant associations with birth weight were observed for freshwater fish and shellfish intake, but not for saltwater fish intake. The health benefits or hazards associated with seafood consumption require further attention to the origin of salt water or fresh water given the reported differences in the storage of polyunsaturated fatty acids and N-3 fatty acids between the two types of fish [27, 28]. The ratio of N-3 to N-6 fatty acids has been shown to be associated with gestation and birth weight [29] and this ratio is approximately four times greater in marine fish in comparison to freshwater fish [27]. However, in a study in Norway, negative associations between fish intake and fetal growth measures were only seen for the consumption of fatty fish but not for the consumption of lean fish [24]. Fatty fish is a well-known important source of dioxins and PCBs, particularly in large marine fish species [30, 31]. Contamination of fish has frequently been suggested as an explanation for the negative associations of fish intake with fetal growth. The differences in the associations between seafood consumption with fetal growth were probably explained by the different effects between beneficial nutrients, mainly DHA, and pollutants present in each meal [32, 33]. Type and species-specific fish consumption remain important considerations in assessing exposure scenarios based on fish consumption.

We also examined seafood consumption with the risk of low birth weight, and the results showed that seafood intake had a protective effect on low birth weight, which is consistent with some earlier findings [21, 24, 34]. Consuming seafood ≥1 time a week during pregnancy was associated with a lower risk of low birth weight. However, some previous studies were not able to quantify the amount of seafood to be consumed to achieve the maximal health benefits. Our study advanced the previous works by examining the weekly amount of total seafood consumption. We found that consuming more than 75 g of seafood/week during pregnancy was associated with a lower risk of low birth weight, which is in line with the recommendations of the Chinese nutrition society dietary guideline for pregnant women [13]. When we examined seafood consumption by type, significant associations with low birth weight were observed for freshwater fish intake, shellfish intake, as well as any two or three kinds of fish intake, but not for saltwater fish. One reason for these discrepant findings might be the sample size of maternal saltwater fish consumption(*n* = 55). For instance, some previous studies have demonstrated that prediction models built using logistic regression in small data sets, might lead to poor predictions that are too extreme and uncertain [35–37]. Other possible explanations relate to more contaminants in saltwater fish [30, 31]. Another possible explanation for the result is that decline in the freshness of the fish leads to lower nutritional quality. Specifically, it takes a long time to transport the frozen marine fish to the inland city of Lanzhou.

Several investigators have reported an increased risk of low birth weight associated with a maternal diet low in marine fish, or even with no association [38]. Differences in findings are partly due to patterns of consumption and type of seafood. In particular, the recommended consumption quantity of seafood varies from study to study and has been shown to range from 85 g to 200 g depending on the type of fish as well as the country of the study [12, 23, 39]. Lanzhou is an inland city with a relatively lower consumption of seafood than coastal cities and is far below the amount (350–525 g/week) recommended by the Chinese nutrition society [13]. In addition to differences in patterns of consumption, the concentrations of nutrients and contaminants in various seafood items depend on whether they are farm-raised or wild-caught, as well as the origin and depth of wild species [40]. In China, freshwater fish primarily comprises cultured carp, a lean fish species with low-fat content. Shellfish are mainly manually raised in mariculture. The Chinese government set very strict standards to limit the concentration of heavy metals [41]. As a result, the concentration of organic pollutants in Chinese farm-raised fish is lower than that in wild fish [42].

In our secondary analysis on the interaction between pre-pregnancy BMI and seafood consumption on birth weight, we found a significant interaction effect among underweight women, but not among overweight women. Abundant evidence has shown that pre-pregnancy BMI was an important factor associated with fetal growth. Specifically, pre-pregnancy underweight was associated with an increased risk of low birth weight, while pre-pregnancy overweight was associated with an increased risk of high birth weight [43, 44]. The interaction between pre-pregnancy BMI and seafood consumption on birth weight among underweight women was consistent with previous studies and suggests that seafood consumption should be encouraged among underweight women to prevent giving birth to low birth-weight babies [45]. However, we didn’t find such an interaction effect among overweight women, which was in contrast with a previous study showing high seafood consumption before pregnancy was positively associated with fetal growth in overweight women [46]. One possible explanation may be the small number of low birthweight babies in the overweight groups, making it impossible to detect statistical significance. Future studies are warranted to further test the interaction effect of pre-pregnancy BMI and seafood consumption on birth weight among overweight women.

In addition, the significant mediation effect of GWG on the association between seafood consumption and birth weight in our secondary analysis was also consistent with a recent study showing similar medication effects of GWG [47]. Inadequate GWG has been well established to be a risk factor for adverse birth outcomes including low birth weight [48], which is especially evident among Asian women who have been shown to have the highest prevalence of insufficient GWG [49]. Our results added further support to the beneficial effect of maternal seafood consumption on birth weight, which can be realized both directly and indirectly through improved GWG. It is thus suggested that pregnant women, especially those with inadequate GWG, should improve their seafood consumption during pregnancy to improve the birth weight of their new babies.

One thing noteworthy is that the rate of gestational diabetes among pregnant women of Lanzhou City in our study was 1.01%, which was slightly lower than the reported 3% in another study conducted in Lanzhou [50]. However, both rates were consistent with the estimated gestational diabetes rate of 1–5% among pregnant women in China but much lower than those reported in other developed countries [51]. Gestational diabetes is a well-known risk factor for higher birth weight [52]. The relatively lower rate of gestational diabetes in our study may be largely explained by the low living standards and young age of the sample. Lanzhou is located in the remote northwest of China with relatively lower socio-economic development. Pregnant women living in Lanzhou are less likely to have high-calorie, high-fat, and low-fiber diets that are characteristic in economically developed areas. As a result, they are less likely to develop gestational diabetes as high-calorie, high-fat, and low-fiber diets are associated with increased risks of gestational diabetes [39]. In addition, studies have shown that the risk of gestational diabetes increases significantly with the increase in age, with the age of over 35 being a significant risk factor for gestational diabetes [39]. In our study, pregnant women older than 35 only accounted for 9.5% of the total population, further explaining the relatively low rate of gestational diabetes in our sample.

The main strength of this study is the relatively large sample of Chinese pregnant women in this study and their high rates of participation and follow-up, which allowed us to extensively collect information on many potential confounding variables. A second highlight is the prospective study design allowing us to collect a range of data from pregnancy to birth outcomes. A third strength is we collected detailed information on a wide variety of maternal characteristics including demographic and nutritional predictors of fetal growth as well as various types of seafood consumption.

Nonetheless, there were some limitations to our study. First, the pregnant women all came from one hospital and about 85% of them completed all routine prenatal visits in the hospital. Our sample may not represent pregnant women in other hospitals or the 15% lost-to-follow-up women with missing information on maternal complications. Second, the assessment of most variables including seafood consumption was based on pregnant women’s self-reports, which may be subject to recall bias and thus limit the methodological strength. However, we also used some metrics such as nutritional intake, infant birth weight, birth length, and head circumference measures to complement this limitation. Third, although careful adjustment for potential lifestyle confounding variables did not appreciably alter the results, we did not control the confounding for other dietary variables. For instance, pregnant women may also get long-chain n-3 fatty acids from other supplements apart from seafood. However, a previous study had shown that adjusting for meat, milk or other dietary factors did not attenuate the associations between seafood consumption and birth weight [24]. Fourth, the assessment of physical activity and total energy intake was based on the whole pregnancy period instead of at different gestational ages. Since physical activity and total energy intake vary considerably during pregnancy, future studies may benefit from collecting such information by different pregnancy stages to get a more accurate assessment. Finally, we did not have the biomarker for chemical exposures, such as PCBs, and Hg. Thus, we could not estimate contaminant intake. Future studies should assess biomarkers of environmental contaminants or nutrients.

## Conclusions

In summary, our study showed that maternal seafood consumption was associated with decreased risk of low birth weight and increased birth weight. This association was mainly driven by freshwater fish and shellfish. Seafood is still an important dietary source of N-3 LCPUFA and proteins for pregnant women and fetuses. These results further corroborate the present dietary recommendation to the Chinese Nutrition Society for pregnant women. However, in our population, 10.7% of pregnant women followed this recommendation. Therefore, our findings suggest that seafood consumption may be strengthened among pregnant women who never consumed or consumed not enough seafood, especially those with underweight pre-pregnancy BMI and with inadequate GWG. Our findings have implications for the future development of intervention programs to improve birth outcomes and fetal growth through the improvement of seafood consumption. This may be realized by the joint efforts of multiple stakeholders involved in seafood and pregnancy care. For instance, healthcare providers can provide more information and education to pregnant women about the beneficial effect of seafood consumption on fetal growth to promote their knowledge, awareness, and behavior in seafood consumption. The local government could collaborate with the seafood market to provide some incentives such as free seafood stamps to encourage seafood purchase and consumption among pregnant women.

## Data Availability

Data described in the manuscript will be made available upon request application to Wang Jianqin, email:wzhang2021@lzu.edu.cn.
